# Oral Lesions and Hematology

**DOI:** 10.4274/tjh.2014.0381

**Published:** 2015-02-15

**Authors:** İrfan Yavaşoğlu

**Affiliations:** 1 Adnan Menderes University Faculty of Medicine, Department of Hematology, Aydın, Turkey

**Keywords:** Oral lesions, S. aureus, Hematology

## TO THE EDITOR

The letter entitled “A Rare Cause of Recurrent Oral Lesions: Chediak-Higashi Syndrome”, written by Karabel et al. and published in a recent issue of your journal, was quite interesting [1]. Here we would like to emphasize some relevant points.

A number of systemic diseases, including hematologic disorders, do have manifestations in the orofacial region. Although non-pathognomonic, these manifestations may often represent the initial sign of the underlying hematopoietic disease. Oral ulcers are often idiopathic (recurrent aphthous stomatitis). However, oral ulcers may be findings of gluten enteropathy, inflammatory bowel disease, or Behçet’s disease. In addition, the cause may be deficiency of vitamin B12/folate/iron in 1 in 4 cases [2]. In particular, such lack of uptake may be the cause in patients who present with mental retardation. Therefore, mean corpuscular volume is important. Chediak-Higashi syndrome usually presents in infancy or early childhood; infections involving the lungs, skin, and mucous membranes are commonly encountered. Dental caries and periodontal disease are also common. The most frequent offending organism is S. aureus. Prophylactic trimethoprim/sulfamethoxazole may be useful. Ascorbic acid (200 mg/day) can also be given [3].
